# Evaluation of antioxidant, diuretic, and wound healing effect of Tulkarm honey and its effect on kidney function in rats

**DOI:** 10.14202/vetworld.2018.1491-1499

**Published:** 2018-10-25

**Authors:** Hamada Imtara, Noori Al-Waili, Meryem Bakour, Wail Al-Waili, Badiaa Lyoussi

**Affiliations:** 1 Laboratory of Physiology, Pharmacology and Environmental Health, Faculty of Sciences, Dhar El Mehraz, BP 1796 Atlas, University Sidi Mohamed Ben Abdallah, Fez 30 000, Morocco; 2 Department of Nephrology and Hypertension, NY Medical Care for Nephrology, New York, USA

**Keywords:** furosemide, honey, kidney, Madecassol, wounds

## Abstract

**Aim:**

The composition and activity of honey depend on its floral origin. Honey collected from Tulkarm was evaluated for physicochemical property and antioxidant content as well as a diuretic and wound healing activity. Its effect on kidney function was evaluated and compared with furosemide.

**Materials and Methods:**

Honey was collected in Tulkarm, Palestine, and its phenol, flavones, and flavonol content were assessed. The antioxidant activity was determined with the use of colorimetric assays, 1,1-diphenyl-2-picrylhydrazyl, ferric reducing antioxidant power, and 2,2-azino-bis(3-ethylbenzothiazoline-6-sulfonic acid). Two sets of experiments were conducted. First experiment: 18 rats were used for the evaluation of diuretic activity of honey. The rats received either honey or furosemide. Renal function test, uric acid, and serum and urine electrolytes assay were performed. Second experiment: 18 male mice were used to evaluate the wound healing property of honey. Wounds were created on mice skin and treated daily with honey or Madecassol. Measurements of wounds were performed over a period of 12 days.

**Results:**

The physical and chemical parameters of Tulkarm honey are within the limits of the European legislation and fulfilling the criteria described in the standard codex for honey. It contains antioxidant compounds and shows antioxidant activity. Oral honey increased creatinine clearance and urine volume, sodium, and chloride without causing hypokalemia or affecting blood urea, uric acid, or serum creatinine level. The diuretic activity of furosemide was associated with hypokalemia. Topical honey application enhanced wound closure when compared with the Madecassol application.

**Conclusion:**

The study is the first to report that honey collected from Tulkarm has a considerable diuretic effect without affecting serum electrolytes or kidney function test and exhibits strong antioxidant activity and wound healing property.

## Introduction

Data have shown that honey has antibacterial, antiviral, antiparasitic, antimutagenic, anti-inflammatory, anticancer, and antioxidant activities, and it has potential in the improvement of wounds, ulcers, diabetes, hypertension, and cardiovascular disease [1-10]. Honey has been mentioned in Holy books, the Talmud, both the old and new testaments of the Bible, and the Holy Quran as a healer of diseases. In the Surat Al-Nahel (The Bee) it says: *And your LORD taught the bee to build its cells in mountains, on tree and in men’s habitations, then to eat of all the fruits of the earth and find with skill the spacious paths of its LORD, their issues from within their bellies, a drink of varying colors, wherein is healing for men, verily in this is a sign for those who give though*t.

Studies have reported the beneficial effect of honey in kidney function and kidney pathology, particularly in diabetes. It has been found that in normal individual’s oral honey collected in the United Arab Emirates increases urinary nitrite excretion, free water clearance, filtered sodium, and creatinine clearance. Furthermore, honey decreases urinary excretion of prostaglandins (PG) F2 alpha, thromboxane B2, and PG E2. On the contrary, artificial honey reduces urinary nitrite and raises urinary PG concentration [7]. Furthermore, carob honey collected in Morocco increases urine output and creatinine clearance during the acute or chronic mode of administration [8]. Interestingly, honey ingestion did not cause hypokalemia in spite of increased urine potassium and urine volume. In kidney diseases, Moroccan carob honey has a protective effect against lead-induced toxicity in rats; it ameliorated the elevation of serum creatinine and blood urea nitrogen induced by lead [9]. In addition, it was demonstrated that honey collected in the United Arab Emirates can protect liver and kidney during acute blood loss, food restriction, and after carbon tetrachloride intoxication [10-14]. Other studies found that honey from different regions showed a protective effect in cisplatin, carbon tetrachloride, and cyclophosphamide-induced kidney toxicity [15-17]. Honey treatment improved the kidney function, increased antioxidant enzymes, and decreased lipid peroxide levels and improved the morphology of the kidney cells of the melamine-fed rats [18]. Pre-treatment with Sidr honey and silymarin before the administration of CCl4 significantly prevented the increase of the serum levels of liver enzyme and reduced oxidative stress. Sidr honey has a significant lipid-lowering effect and it protected liver and kidney lesions [17]. In diabetic rats, administration of honey reduced mesangial matrix expansion and thickening of glomerular basement membrane [19].

Honey bees (*Apis mellifera*) collected honey from different plants, and the composition of honey depends on the type of plant, climate, and environmental conditions. Regarding the complementary medicine use in certain areas of Palestine, it was reported that 72.8% of participants in the northern part of Palestine have used at least one type of complementary medicine. It was concluded that alternative medicine utilization in Palestine is very common which includes honey [20]. However, few studies have been published so far regarding the quality and functional properties of honey collected in Palestine. One of these studies showed that ingestion of Palestinian honey induces spermatogenesis in rats by increasing epididymal sperm count, increases the relative weight of the epididymis and lactate dehydrogenase activity, and reduces sorbitol dehydrogenase activity [21]. Furthermore, the volatile compounds as markers in Palestinian honey have been studied [22,23].

The objectives of the present study included evaluation of the quality of honey through the determination of physical, chemical, and antioxidant property, the diuretic effect of honey compared to furosemide, the effect of honey on kidney function test, and the wound healing effect of honey compared to Madecassol. This is the first study to explore these variables in Tulkarm honey collected in Palestine. Tulkarem is a coastal city famous by citrus and avocado trees.

## Materials and Methods

### Ethical approval

The care and handling of rats and mice were in accordance with internationally recognized standards guidelines for the use of animals. The protocol was approved by the Institutional Committee on Animal Care following the French Technical Specifications for the Production, Care and Use of the Laboratory Animals, University Sidi Mohamed Ben Abdellah, Faculty of science, Dhar Mehrez, Fez, Morocco.

### Physicochemical analysis

Honey sample (Multifloral honey) was collected from the city of Tulkarm, Palestine. Analysis of sample was carried out in triplicate for each test to determine the physicochemical properties (moisture content, total ash content, electrical conductivity, pH, free acidity, lactone acidity, proline, honey color, and melanoidin content). The measurements were achieved according to the International Honey Commission (IHC) [24]. The color was measured according to the method described earlier [25]. The degree of browning measured through the absorbance of samples between 420 and 450 nm is used to assess the extent to which the Maillard reaction occurs in foods, constituting one way to estimate the late-stage of the Maillard reaction products: The melanoidins [25].

### Analysis of antioxidant activity

The antioxidant activity of honey was determined by different methods such as total antioxidant capacity, the radical scavenging activity of the honey solution toward 1,1-diphenyl-2-picryl hydrazyl (DPPH) free radical and 2, 2-azino-bis 3-ethylbenzothiazoline-6-sulfonic acid (ABTS*^+^), and ferric reducing antioxidant power [26-30]. All the assays were carried out in triplicate, and average values were considered. Several concentrations of samples were made, and the percentage inhibition and inhibitory concentration (IC_50_) (concentration of sample able to scavenge 50% of ABTS*^+^ or DPPH radicals) were determined.

### Analysis of phenol, flavones, and flavonol

The total phenolic content was determined by the use of the Folin–Ciocalteu assay and total phenol content was expressed as mg gallic acid equivalents (GAEs)/100 g [26]. The total flavones and flavonol content were determined, and the results were expressed as mg quercetin equivalents (QEs)/100 g [31]. The antioxidant activities of honey samples were determined by the phosphomolybdenum method, and they were expressed relative to ascorbic acid (mg AAE/100 g) [32].

### Experimental animals

Eighteen Wistar rats weighing between 200 and 240 g were used for the evaluation of diuretic activity and 18 male mice weighing between 28 and 35 g were used to observe the wound healing effect. The animals were obtained from the animal house breeding center, Faculty of Sciences, Dhar Al-Mahraz, Fez, and were housed under normal environmental conditions (25±1°C 55±5% humidity and 12 h/12 h cycle light/dark). The animals were allowed free access to tap water and standard laboratory food.

### Reference drug

Furosemide (Lasilix, Pharma 5, Morocco) was used as a reference drug. Madecassol cream (Bayer laboratory, France) was used as reference drug for wound healing. One gram of Madecassol, 1% cream, contains 10 mg *Centella asiatica*; (reconstituted titrated dry extract containing asiaticoside; madecassic and asiatic acids)

### Biochemical methods

Blood samples were collected in capillary tubes containing ethylenediaminetetraacetic acid by retro-orbital puncture under light diethyl ether anesthesia. After centrifugation at 3500 RPM for 10 min, the plasma was separated and kept at −20°C until analysis. The levels of the blood and urine sodium, chloride and potassium were measured with the use of a spectrophotometer. Kidney function test was performed by estimation of serum creatinine, uric acid, and blood urea nitrogen. Osmolar clearance was determined from plasma osmolality, urinary osmolarity, and urine flow according to the following formula: Osmolar clearance=urinary osmolarity×urine flow/plasma osmolality. Free water clearance was determined from plasma osmolality, urinary osmolarity, and urine flow according to the following formula: Free water clearance=urine volume (ml/min)×(1-urine osmolarity/plasma osmolarity). T_CH2O_ (free water reabsorption) was assessed by C_H2O_ according to the following formula; T_CH2O_ = - (C_H2O_).

### Assessment of diuretic activity

Each animal was placed in an individual metabolic cage for a period of 3 days to adapt to the new conditions before the experiment. The rats were randomly divided into three groups, sex animals each: Group 1 received oral administration of distilled water at a dose of 10 ml/kg.b.wt and served as the control group, Group 2 was treated with an oral dose of 10 mg/kg.b.wt of furosemide, and Group 3 received oral administration of 100 mg/kg.b.wt of the honey. After administration of the interventions, urine samples were collected and measured at 1, 2, 4, 6, and 24 h after the first dose of each intervention. Then after, daily oral doses were administered to three groups of rats for 11 days, and for each rat, 24 h urine was collected daily and its volume was measured. Urinary sodium, chloride, and potassium concentrations were measured in the urine specimens on day 11. Serum sodium, potassium, chloride, uric acid, and creatinine levels, blood urea nitrogen, and urinary creatinine excretion were measured on day 11. Other variables, including plasma and urine osmolarity and free water clearance, were also measured on day 11.

### Effect of honey on wound healing

The mice were anesthetized through intraperitoneal injection of pentobarbital sodium (0.05 mg/g weight), and their dorsal hair was shaved off. A circular full-thickness skin wound (7.4 mm in diameter), which included the panniculus carnosus muscle, was performed on the skin of each mouse. The animals were divided into three group’s six mice each: Group 1 (Control) no treatment; Group 2 (Madecassol group) received a daily application of Madecassol ointment applied directly to the skin; and Group 3 (the honey group) received a daily application of honey applied directly to the wounds. The wound healing was observed on days 1, 3, 7, and 12. The edges of the wounds were traced on polypropylene sheets, and photographs of the wounds were taken every day. The traces on the polypropylene sheets were then captured with a scanner and transferred to the computer using the image processing software (Picture manager) to improve contrast. The area of each wound was calculated using the image analysis software Adobe Illustrator CS5 NA. The wound was treated daily by applying Madecassol. In honey group, the wound was treated daily by applying 0.1 ml of the honey (5 g of honey+1 ml of distilled water).

### Statistical analysis

The results were expressed as mean values±standard error of the mean. Statistical analysis of the data was performed with one-way analysis of variance (ANOVA) followed by Tukey’s multiple comparison test (ANOVA followed by Tukey’s test) (GraphPad Prism, version 6). Significant differences were indicated by p<0.05.

## Results

### The physicochemical parameters and antioxidant content

The physicochemical parameters of honey studied revealed that the pH is 4.70±0.01, free acidity is 42.17±2.08 mEq/kg, lactone acidity is 7.73±1.17 mEq/kg, total acidity is 49.89±1.24 mEq/kg, the percentage of moisture is 17.73±0.115%, electric conductivity is 1153.33±4.51 µS/cm, ash is 0.508%, color is 0.18±0.03, melanoidin is 0.44±0.02, and proline is 448.81±5.06 mg/kg. The classification of the honey depends on the value of Pfund scale (78.77±5.31 mm) indicating that the honey is light amber.

The data showed that the level of polyphenol is 78.79±1.43 mg AGE/kg, flavones and flavonols are 4.47±0.23 mg QE/kg, and total antioxidant capacity is 70.76 ±1.89 mg AAE/g. The antioxidant activities results showed that the IC_50_ is 17.14±1.40 mg/ml for DPPH assay, 3.16±0.70 mg/ml for ABTS*^+^ assay, and 1.32±0.03 mg/ml for ferric reducing antioxidant power.

### Effect of honey on urine and plasma electrolytes and urine volume and osmolarity

Treatment with a single dose of Tulkarm honey increased urine output, which became significantly higher than the water at 4 h after the administration (Table-1). The urine output was increased significantly by honey administration throughout the study period as compared to the water. Furosemide caused early and higher diuresis than honey. Furosemide caused a significant increase in the urine output in all the intervals, and it was more potent than honey.

**Table-1:** Urine volume during 11 days with daily oral administration of the interventions.

Days	Urine volume (ml/24 h)		

	Water	Honey	Furosemide
1	5.67±0.34	6.50±0.29^+^	7.00±0.58^+^
2	6.00±0.58	8.40±0.06^+#^	8.17±0.09^+^
3	5.74±0.21	7.53±1.07^+#^	8.22±0.62^+#^
4	6.78±1.49	8.23±0.79^+#^	9.22±0.40^+#^
5	6.89±1.49	10.40±0.70^+#^	12.00±0.58^+#^
6	7.20±1.11	10.53±0.24^+#^	12.33±0.89^+#^
7	7.17±1.60	11.90±0.26^+#^	13.80±0.42^+#^
8	6.88±0.73	11.96±0.55^+#^	15.67±0.33^+#^
9	6.56±1.09	12.50±0.28^+#^	17.43±0.29^+#^
10	6.78±0.90	13.67±0.34^+#^	18.83±0.17^+#^
11	6.67±1.20	15.00±0.58^+#^	18.67±0.60^+#^

^+^p<0.05 in comparison to water at the same time interval. *p<0.05 in comparison to honey at the same time interval. ^#^p<0.05 in comparison to day 1 in each group

A significant increase in the urinary volume was observed in all time intervals after the administration of honey or furosemide (Table-2). Furosemide was more potent than honey on days 7-11.

**Table-2:** Effect of oral administration of honey and furosemide on urine volume and electrolyte excretion on day 11.

Interventions	Urine volume (ml/24 h)	Urine electrolytes			Saluretic index			Diuretic index	Lipschtiz value	Ratio Na^+^/K^+^
	
		Na^+^	K^+^	Cl^−^	Na^+^	K^+^	Cl^−^			
Water	6.6±1.20	169±0.5	243±0.64	242±0.57	1	1	1	1	-	0.69
Furosemide	15.00±0.58^+^	278±3.7^+^	308±3.0^+^	281±1.1^+^	1.65	1.26	1.16	2.8	-	0.90
Honey	18.6±0.6^+^	233±3.7^+^*	245±3.2*	298±1.5^+^*	1.38	1.00	1.23	2.25	0.80	0.95

+ p<0.05 in comparison to water.

* p<0.05 in comparison to furosemide

**Figure-1:**
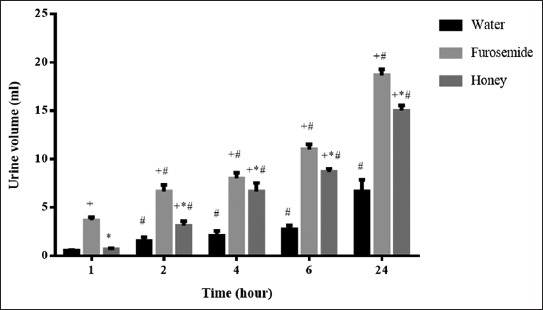
Urine volume (ml) after a single oral dose of each intervention. ^+^p<0.05 in comparison to water in each time interval. *p<0.05 in comparison to furosemide in each time interval. ^#^p<0.05 in comparison to urine volume at 1^st^ h with the use of the same intervention.

Honey caused a significant increase in the urine concentration of sodium and chloride, while the concentration of potassium did not change significantly (Figure-1). Furosemide caused a significant increase in the urine concentration of sodium, potassium, and chloride. The Na^+^/K^+^ ratio for furosemide and honey was 0.90 and 0.95, respectively, as compared to 0.69 for the control. Furosemide caused significantly higher sodium and potassium urinary excretion than honey.

**Figure-2:**
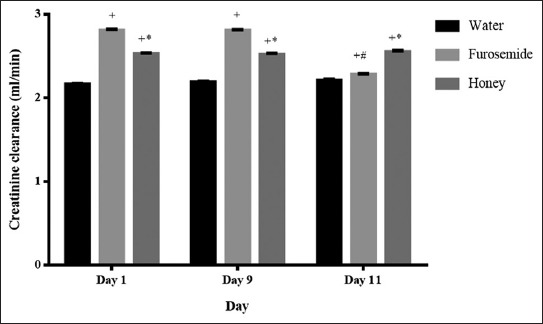
Effect of daily oral administration of honey or furosemide on creatinine clearance. ^+^p<0.05 in comparison to water at the same time interval. *p<0.05 in comparison to furosemide at the same time interval. ^#^p<0.05 in comparison to day 1 in each group.

Honey did not cause significant changes in the plasma electrolytes, creatinine, uric acid, and blood urea nitrogen level. However, it caused a significant increase in the creatinine clearance during all the time intervals (Table-3 and Figure-2). Furosemide caused a significant decrease in the plasma potassium and chloride level in comparison with the water group. It also increased creatinine clearance in all time intervals, and in comparison to honey, it was less potent on day 11.

**Table-3:** Effect of honey and furosemide on plasma electrolytes and kidney function test on day 11.

Interventions	Creatinine (mg/dl)	Uric acid (mg/dl)	Urea (mg/dl)	Na^+^mmol/l	K^+^mmol/l	Cl^−^mmol/l
Water	0.4±0.05	11.2±0.08	18±3	163±1.2	4.5±0.01	100±1
Furosemide	0.38±0.02	11.5±1.3	15±1	160±2.3	3.8±0.07^+^	85±7.7^+^
Honey	0.41±0.04	12±1.5	15±1	161±14	4.4±0.12	90±5.1^+^

^+^p<0.05 in comparison to water. *p<0.05 in comparison to furosemide

Honey did not cause a significant change in the plasma osmolarity. However, it caused significant increase in the urine osmolarity, osmolar clearance, and free water clearance compared to the control (Table-4). Furosemide increased free water clearance and osmolar clearance and decreased urine osmolarity.

**Table-4:** Effect of daily administration of honey and furosemide on plasma osmolarity; urine osmolarity; osmolar clearance and clearance of free water on day 11.

Variables	Water	Honey	Furosemide
Urinary flow (µl/min)	4.6±2.00	10.4±2.80^+^	12.9±3.10^+^*
U_osm_ (mOsm/kgH_2_O)	825.8±12.1	957.2±3.89^+^	1172±13.34^+^*
P_osm_ (mOsm/kgH_2_O)	324.6±7.5	322.6±6.9	321.3±8.8
C_osm_ (µl/min)	11.7±2.67	30.9±3.50^+^	47.27±2.20^+^*
C_H20_ (µl/min)	−10.2±1.30	−29.5±2.34^+^	−49.4±4.29^+^*
T_cH2O_ (µl/min)	10.2±2.20	29.5±3.90^+^	49.4±2.80^+^*

^+^p<0.05 in comparison to water. *p<0.05 in comparison to honey

### Effect of honey on wound healing

**Figure-3:**
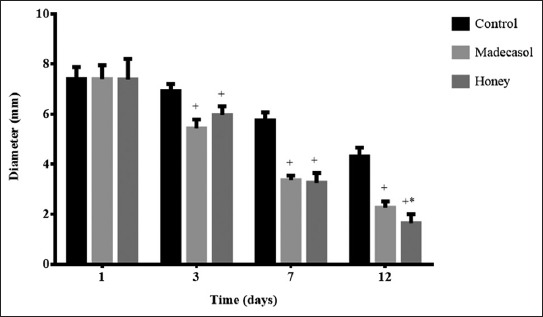
Diameter (mm) of the wounds observed at each time interval following treatment by honey or Madecasol and compared with the control (no treatment). ^+^p<0.05 in comparison to the control. *p<0.05 in comparison to Madecasol.

**Figure-4:**
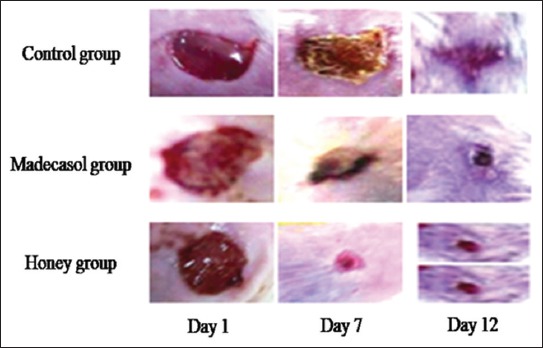
Macroscopic changes during wound healing with the use of various interventions.

There was no significant difference in the diameter of the wounds treated with honey on day 1 and 3 as compared to the control (Figure-3). However, on days 7-12, the wounds treated with honey or Madecassol showed significantly smaller size than wounds treated with the control (p<0.05). On day 12, the size of wounds treated with honey was less than sized of wounds treated with Madecassol, but the difference was statistically insignificant. Figure-4 demonstrated the macroscopical changes occurred during the treatment with interventions.

## Discussion

The physicochemical analysis of the honey revealed that the pH value and the free acidity value were within the limits of European legislation and indicated the absence of undesirable fermentation [33,34]. The pH is acidic, and it is similar to honey pH found in other studies [35-38]. The determined acidity of honey is due to the presence of organic acids and inorganic ions.

The value of free acidity and lactone acidity is within the range of values of Moroccan honey samples that showed the values of free acidity, lactone acidity, and total acidity ranged from 11.0 to 42.5, 4.0 to 16.50, and 17.50 to 59.0, respectively [38]. The total acidity is the sum of free acidity and lactonic acidity. It was similar to the multifloral Portuguese honey acidity and relatively higher than multifloral honey acidity from Morocco carob honey (49.89±1.24 vs. 33.18 mEq/kg) [25,27].

Moisture percentage of the Tulkarm honey is below the average limit of ≤20% for blossom honey [35]. The moisture value of Palestinian honey is in accordance with values recommended by the IHC, in 2009, and the Codex Standard for Honey 2001 and was similarly compared with the values obtained for carob honey from Italy and Portugal and Morocco [27,36,38].

The percentage of ash in the honey was within the limits of Codex Alimentarius. The percentage of ash value depends on the mineral contents. Honey collected in Italy and Turkey showed almost similar ash content [36,39]. The ash content in various honey samples collected in Morocco ranged between 0.13% and 0.69%, and the largest value was detected in carob honey from Taounate Region (0.69±0.01%) [38].

The electrical conductivity is a useful parameter to determine the botanical origin of honey and the difference between nectar and honeydew [33]. The electrical conductivity depends on the mineral content of the honey. The value found in the honey sample was higher than the maximum limits defined by the Codex Alimentarius Commission [40]. It was found that the electrical conductivity of honey samples from Morocco varied in the range of 0.36 mS/cm-1.35 mS/cm [38]. High values were found in carob honey from Taounate Region (1.35±0.03 mS/cm).

Phenolics compounds are important bioactive molecules found in honey. The total phenol and flavonoid (flavones and flavonols) contents of eight different Morrocan honey samples varied from 755.2 to 2452.2 mg GAE/kg and 22.6 to 47.9 mg QE/kg, respectively [38]. Therefore, the flavones and flavonols found in Tulkarm honey were higher than the range of eight Moroccan honeys while polyphenols were within the range of the Moroccan honeys. Furthermore, the phenolic content of Tulkarm honey samples was higher than that reported in honey from Yemen (751.3-2462.1 mg/kg), Brazil (611.6-1113.7 mg GAE/kg), India (490-980 mg GAE/kg), Serbia (274.4-614.2 mg GAE/kg), and Algeria (158.4-616.3 mg/kg) [41-44].

The IC_50_ value of ABTS^*+^ was 3.16±0.70 mg/ml; this value is within four multifloral Moroccan honeys and less than two Portugal multifloral honeys [25,27]. The IC_50_ value of DPPH was 17.14±1.40 mg/ml which is higher than Polish multiforal honeys and less than Slovenian honey [30,45].

It was found that the total antioxidant power of carob honey varied from 35.03 mg AAE/g to 60.94 mg AAE/g, which is less than that of Tulkarm honey [38]. The concentration of Tulkarm honey required to inhibit 50% of DPPH was higher than the concentration of carob honey (12.54 mg/ml and 23.10 mg/ml), Serbian honey (11.16-48.48 mg/ml), and Turkish honey (12.56-152.40 mg/ml) [38,41,46]. The reducing power of Tulkarm honey was lower than reducing the power of carob honey (1.87 mg/ml-4.40 mg/ml) [38].

Furosemide increases urinary output and urinary excretion of sodium, potassium, and chloride by inhibiting Na^+^/K^+^/2Cl^−^ symporter (cotransporter system) in the thick ascending limb of the Loop of Henley [47]. The study reveals that honey demonstrates a significant diuretic activity. Honey causes a significant increase in urine concentration of sodium and chloride, while the concentration of potassium did not change significantly. However, furosemide causes a significant increase in the urine concentration of sodium, potassium, and chloride as well as hypokalemia. Therefore, the action of honey is almost similar to thiazide diuretics, which increase the excretion of sodium and chloride by inhibition of Na^+^/Cl^−^ symporter (cotransporter system) in the distal convoluted tubule [47]. Interestingly, contrary to thiazide and furosemide, honey did not cause hypokalemia that is a common side effect of loop or thiazide diuretics. This might be due to high levels of potassium presents in honey [5,38]. It was found that honey increases urine excretion of nitric oxide, and consequently, it was proposed that the diuretic effect of honey, partly, is due to increased urinary nitric oxide concentration [7]. Honey collected from Palestine shares the same diuretic activity with honey samples collected from Morocco and United Arab Emirates [7,8].

Several studies showed that flavonoids have been linked to the diuretic effects [48,49]. This might explain, in part, the diuretic activity of honey. The glucose and fructose in honey might cause diuresis. However, we found that artificial honey causes insignificant changes in the urine volume in healthy individuals [7].

Regarding the wound healing potential of the Palestinian honey, the macroscopic examination of the wound reveals a significant decrease in the diameter of the wounds treated by honey as compared with wounds treated with Madecassol. The healing property of honey is mainly due to its anti-inflammatory, antioxidant, and antibacterial activity [50,51]. Furthermore, its acidity and hyperosmolarity, as well as the bioactive molecule such as flavonoids, hydrogen peroxide, glucose oxidase, gluconic acid, and MGO, play a major role in its activity [50,52,53]. The wound healing activity could also be due to inhibition of PG, stimulation of nitric oxide and modulating the release of anti-inflammatory cytokines and growth factors by monocytes [54,55]. Madecassol was used to compare its effect with honey. It is commonly used products for burn, skin wrinkles, and wound management [56,57]. Their most beneficial effect appears to be the stimulation of maturation of scar by the production of Type I collagen and resulting decrease in the inflammatory reaction and myofibroblast production.

## Conclusion

**Figure-5:**
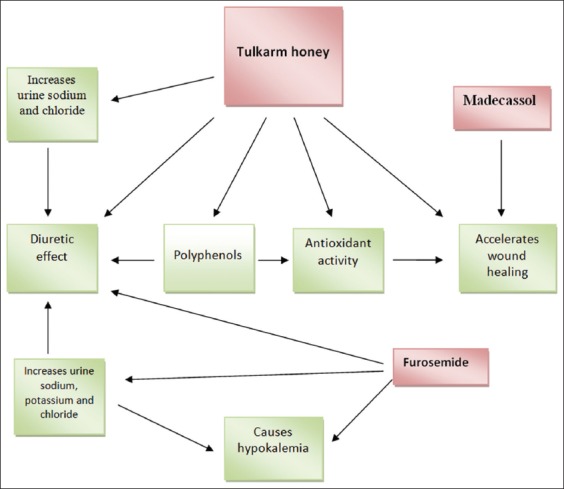
The effect of Tulkarm honey on wound healing and diuresis.

The findings showed, the physicochemical characterization, antioxidant and wound healing property, and diuretic effect of honey collected from Tulkarm, Palestine (Figure-5). Tulkarm honey contains phenols, flavonols, and flavones, and it exhibits considerable antioxidant activity. It also has a diuretic effect without affecting serum electrolytes or kidney function test. As compared to Madecassol, Tulkarm honey showed significant wound healing properties, which might be due to its antioxidant activity, acidity, nutritional content, or other factors such as anti-inflammatory and antimicrobial activity.

## Authors’ Contributions

HI, MB, and BL involved in the design and conception of the experimental protocols and participated in the experimental work; NA and WA wrote the paper and prepared for publication, conducted the statistical analysis of the work, and did acquisition and interpretation of the data. All authors read and approved the final manuscript.
